# Restricted Localization of Photosynthetic Intracytoplasmic Membranes (ICMs) in Multiple Genera of Purple Nonsulfur Bacteria

**DOI:** 10.1128/mBio.00780-18

**Published:** 2018-07-03

**Authors:** Breah LaSarre, David T. Kysela, Barry D. Stein, Adrien Ducret, Yves V. Brun, James B. McKinlay

**Affiliations:** a Department of Biology, Indiana University, Bloomington, Indiana, USA; University of Washington

**Keywords:** intracytoplasmic membrane, light harvesting, purple nonsulfur bacteria, spatial organization

## Abstract

In bacteria and eukaryotes alike, proper cellular physiology relies on robust subcellular organization. For the phototrophic purple nonsulfur bacteria (PNSB), this organization entails the use of a light-harvesting, membrane-bound compartment known as the intracytoplasmic membrane (ICM). Here we show that ICMs are spatially and temporally localized in diverse patterns among PNSB. We visualized ICMs in live cells of 14 PNSB species across nine genera by exploiting the natural autofluorescence of the photosynthetic pigment bacteriochlorophyll (BChl). We then quantitatively characterized ICM localization using automated computational analysis of BChl fluorescence patterns within single cells across the population. We revealed that while many PNSB elaborate ICMs along the entirety of the cell, species across as least two genera restrict ICMs to discrete, nonrandom sites near cell poles in a manner coordinated with cell growth and division. Phylogenetic and phenotypic comparisons established that ICM localization and ICM architecture are not strictly interdependent and that neither trait fully correlates with the evolutionary relatedness of the species. The natural diversity of ICM localization revealed herein has implications for both the evolution of phototrophic organisms and their light-harvesting compartments and the mechanisms underpinning spatial organization of bacterial compartments.

## IMPORTANCE

Many bacteria organize their cellular space by constructing subcellular compartments that are arranged in specific, physiologically relevant patterns. The purple nonsulfur bacteria (PNSB) utilize a membrane-bound compartment known as the intracytoplasmic membrane (ICM) to harvest light for photosynthesis. It was previously unknown whether ICM localization within cells is systematic or irregular and if ICM localization is conserved among PNSB. Here we surveyed ICM localization in diverse PNSB and show that ICMs are spatially organized in species-specific patterns. Most strikingly, several PNSB resolutely restrict ICMs to regions near the cell poles, leaving much of the cell devoid of light-harvesting machinery. Our results demonstrate that bacteria of a common lifestyle utilize unequal portions of their intracellular space to harvest light, despite light harvesting being a process that is intuitively influenced by surface area. Our findings therefore raise fundamental questions about ICM biology and evolution.

## INTRODUCTION

**FIG 1:**
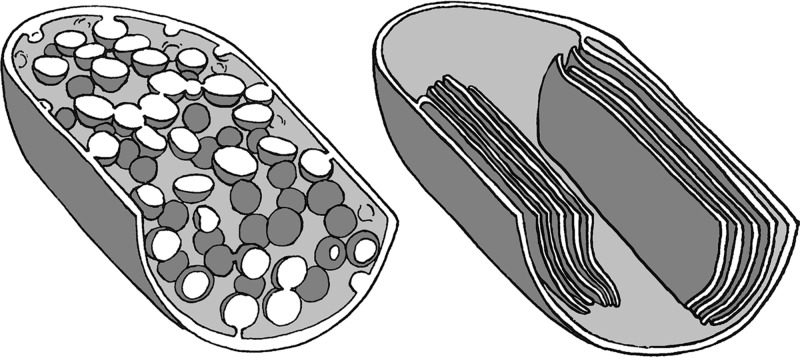
Illustration of vesicular (left) and lamellar (right) ICM architectures. Quarter-cell illustrations (not to scale) are based on published EM images of Rba. sphaeroides (16) and Rps. palustris (7), respectively.

Diverse bacteria utilize protein- or membrane-bound subcellular compartments that afford specific metabolic capabilities (1). A prominent example of such compartments is the intracytoplasmic membrane (ICM) of purple nonsulfur bacteria (PNSB). ICMs (also known as chromatophores) house the proteins and pigments required for photosynthesis (2), akin to the thylakoid membranes of cyanobacteria and plant chloroplasts. ICMs originate from invaginations of the cytoplasmic membrane (CM) (3), remain partially or fully contiguous with the CM following their elaboration (4–8), and are presumed to enhance the efficiency of light capture and energy transformation (5, 9, 10). Amid their comparable origin and function, ICMs exhibit species-specific architectures, ranging from vesicles to lamellae (Fig. 1) (3). ICM architectures are currently understood to result from intermolecular interactions between components of the photosynthetic machinery (11–14).

ICMs have been used for decades as model systems for studying membrane biogenesis and photochemistry (2, 3, 15), but much remains to be learned about how these relatively large intracellular structures are spatially organized (i.e., localized) within a cell. This knowledge gap in spatial organization of ICMs is presumably because past studies have had to rely on biochemical analysis of bulk ICM and electron microscopy (EM) of a small number of cells. These EM images have yielded at least one consistent organization-related observation though, which is that a correlation exists between ICM architecture and ICM localization with regard to the cytoplasmic interior; while ICM vesicles can extend throughout the cytoplasm (4, 11, 16), ICM lamellae appear to be confined to the cytoplasmic periphery (Fig. 1) (5–7, 17–19). However, beyond this correlation, the spatiotemporal coordination of ICM placement remains largely unexplored, particularly considering that while PNSB comprise more than 20 genera (2), most ICM studies have focused on only a handful of species of *Rhodobacter*, *Rhodospirillum*, and *Rhodopseudomonas*. PNSB are phylogenetically and physiologically diverse (2), and thus it would be premature to assume that all species coordinate ICMs equivalently.

Bearing this in mind, here we characterized spatial organization of ICMs using a combination of fluorescence and electron microscopy and quantitative computational analysis of fluorescence microscopy images. By surveying 14 diverse PNSB species, we established that ICMs are subject to differential spatial organization that goes beyond confinement of lamellar ICMs to the cytoplasmic periphery. Specifically, ICM lamellae were differentially localized with regard to the long axis of the cell, with species of several genera restricting ICMs to discrete, nonrandom sites near cell poles. We further established that ICM localization patterns are partially independent of both ICM architecture and species phylogeny.

## RESULTS

### BChl autofluorescence is a noninvasive tool for visualizing ICMs in live cells.

We first set out to develop a method for visualizing the ICM-residing photosynthetic machinery (photosystems) using the natural fluorescence (autofluorescence) of bacteriochlorophyll (BChl); this approach emulates the use of chlorophyll autofluorescence to visualize photosynthetic components within plant chloroplasts and cyanobacteria (20, 21). We deemed BChl to be a suitable ICM marker for several reasons. First, BChl only accumulates under conditions that stimulate ICM and photosystem synthesis (22, 23). Second, BChl is an obligate component of PNSB photosystems and is thus essential for ICM synthesis (3). Finally, BChl exists almost entirely within photosystems, with very little free in the cytoplasm (24), and these photosystems predominantly reside within ICMs (7, 25–27).

**FIG 2:**
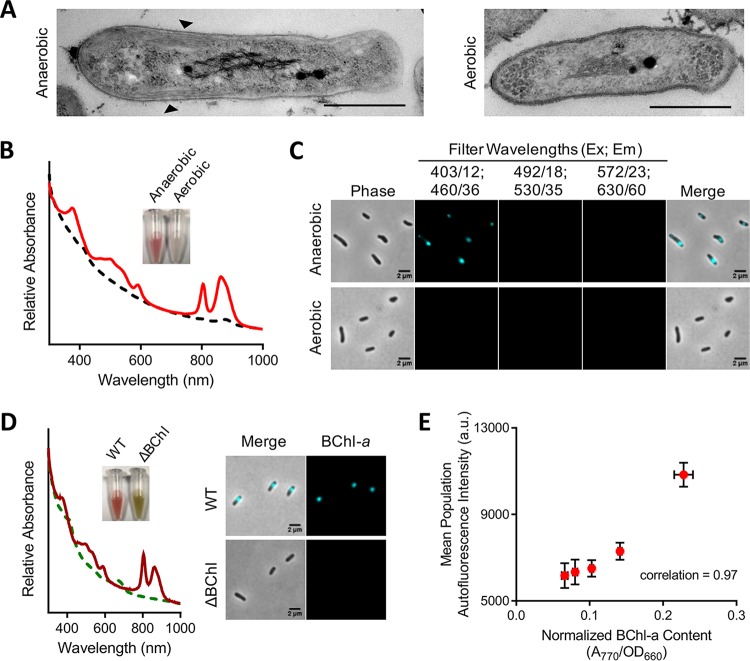
Bacteriochlorophyll (BChl) autofluorescence as a proxy for ICMs in live cells. (A) Electron microscopy images of wild-type (WT) Rps. palustris CGA009 cells grown in PMsuccYE either anaerobically in 8 µmol s^−1^ m^−2^ light (left) or aerobically in darkness (right). Scale bars, 500 nm. Arrowheads indicate ICMs. (B) Spectral analysis of WT Rps. palustris cells grown anaerobically (solid red line) or aerobically (dashed black line) as described for panel A. (Inset) Photograph of concentrated cells grown under each condition. (C) Phase-contrast and fluorescence microscopy images of cells from cultures used in panel B using the indicated filter sets (shown as excitation center [Ex]/range; emission center [Em]/range). Image contrast and brightness are equivalent between fluorescence panels. (D) Spectral analysis (left) and microscopy images (right) of WT (solid dark red line) and BChl-deficient (ΔBChl) (dashed green line) Rps. palustris, each grown by anaerobic respiration with N_2_O in darkness. BChl-*a* fluorescence was detected using the 403/12, 460/36 filter set. Image contrast and brightness are equivalent between fluorescence panels. (Left inset) Photograph of concentrated cells of each strain. (E) Correlation analysis of BChl-*a* content and cellular BChl fluorescence intensity. Points represent mean population cellular BChl fluorescence intensity of independent cultures of WT Rps. palustris grown anaerobically in PMsuccYE at light intensities between 8 and 60 µmol s^−1^ m^−2^. BChl fluorescence (background-corrected mean cellular intensity) was measured using MicrobeJ. Error bars represent the standard deviation (SD) from 3 technical replicates sampled over 2 days. Cell counts for technical replicates are as follows: 8 µmol s^−1^ m^−2^, *n =* 251, 869, 1,337 cells; 20 µmol s^−1^ m^−2^, *n =* 382, 930, 1,535 cells; 32 µmol s^−1^ m^−2^, *n =* 392, 876, 1,420 cells; 50 µmol s^−1^ m^−2^, *n =* 421, 786, 1,101 cells; 60 µmol s^−1^ m^−2^, *n =* 300, 973, 1,419 cells.

We tested the hypothesis that BChl fluorescence could be used to visualize ICMs using the model PNSB species Rhodopseudomonas palustris. Like most PNSB, Rps. palustris synthesizes ICMs only under low-oxygen conditions that allow anoxygenic photosynthesis (22, 26, 28); cells grown anaerobically in light (phototrophically) contain ICMs, whereas those grown aerobically in darkness (chemotrophically) do not. In accord with oxygen-regulated ICM synthesis, aerobically grown Rps. palustris cells lacking ICMs were deficient for pigmentation, whereas phototrophically grown cells containing ICM were pigmented and exhibited characteristic photosystem-associated absorbance peaks (Fig. 2A and B). When we examined Rps. palustris by epifluorescence microscopy, phototrophic cells exhibited autofluorescence detected using a DAPI filter set (excitation [Ex], 403/12; emission [Em], 460/36) (Fig. 2C). No autofluorescence was detected using several other common filter sets (Fig. 2C). In contrast to phototrophic cells, there was no autofluorescence detected in aerobic cells using any of these filter sets (Fig. 2C). Thus, autofluorescence was specific to ICM-containing cells.

We next verified that autofluorescence was derived from BChl using a mutant lacking BChl. As BChl is essential for phototrophic growth, we exploited a growth condition under which ICMs are induced but not required (23). Specifically, we grew Rps. palustris by anaerobic respiration in darkness using N_2_O as the terminal electron acceptor (29). Under these conditions, the absence of O_2_ prompts ICM synthesis, but energy is generated via respiration with N_2_O, and thus growth of a BChl-deficient mutant (ΔBChl) was comparable to that of the wild type (WT) (see Fig. S1 in the supplemental material). WT Rps. palustris cells grown by anaerobic respiration exhibited autofluorescence, whereas ΔBChl cells did not (Fig. 2D). Separately, we also examined whether other photosystem-associated pigments, carotenoids, were responsible for autofluorescence by examining a Δ*crtI* mutant that produces BChl but not carotenoids (see Fig. S2 in the supplemental material) (30). Unlike the ΔBChl mutant, Δ*crtI* mutant cells still exhibited autofluorescence, though at a lower intensity than WT cells (Fig. S2). The decreased intensity was not surprising given that carotenoids, while not essential for ICM synthesis, contribute to normal photosystem assembly (18, 30–32) and thereby affect both BChl levels and the local environment of BChl, which is known to influence BChl spectral properties (9, 11, 18, 33). While it is possible that carotenoids contribute to autofluorescence, our data demonstrate that carotenoids are not necessary for autofluorescence.

10.1128/mBio.00780-18.1 FIG S1  During anaerobic respiration, synthesis of photosynthetic machinery is induced but is dispensable for growth. (A) Growth curves of WT (dark red circles) and ΔBChl mutant (green squares) Rps. palustris grown by anaerobic respiration with N_2_O in darkness. Growth was monitored at OD_630_ rather than OD_660_ due to the absorbance peak near 660 nm in the ΔBChl mutant (see Fig. 2D). (B) BChl content of WT and ΔBChl cells, confirming that deletion of *bchXYZ* abolishes BChl synthesis. Error bars show SD (*n =* 3). Download FIG S1, TIF file, 0.2 MB. Copyright © 2018 LaSarre et al. 2018 LaSarre et al. This content is distributed under the terms of the Creative Commons Attribution 4.0 International license.

10.1128/mBio.00780-18.2 FIG S2  Focal BChl fluorescence persists in the absence of carotenoids. (A) Spectral analysis of WT (red) and Δ*crtI* mutant (blue) Rps. palustris strains grown in anaerobic PMsuccYE in 20 µmol s^−1^ m^−2^ light, confirming loss of carotenoid synthesis in the mutant. (Inset) Photograph of concentrated cells of each strain. (B) Microscopy images of WT and Δ*crtI* cells from cultures used in panel A. Download FIG S2, TIF file, 0.8 MB. Copyright © 2018 LaSarre et al. 2018 LaSarre et al. This content is distributed under the terms of the Creative Commons Attribution 4.0 International license.

We also evaluated the relationship between cellular BChl levels and autofluorescence intensity. In PNSB, BChl levels directly correlate to photosynthetic machinery and ICM abundance (4, 7, 34, 35), and these levels are inversely proportional to light availability (22). We grew Rps. palustris at different light intensities and compared BChl content to autofluorescence intensity. In agreement with our initial hypothesis, we observed a direct correlation between cellular BChl content and cellular autofluorescence intensity (Fig. 2E). Given this correlation, and in combination with the prior data, we conclude that autofluorescence is derived from BChl and infer that BChl fluorescence can be used as a noninvasive marker for ICM localization in live PNSB.

### ICMs are spatially restricted in Rps. palustris in a pattern that promotes ICM inheritance upon cell division.

**FIG 3:**
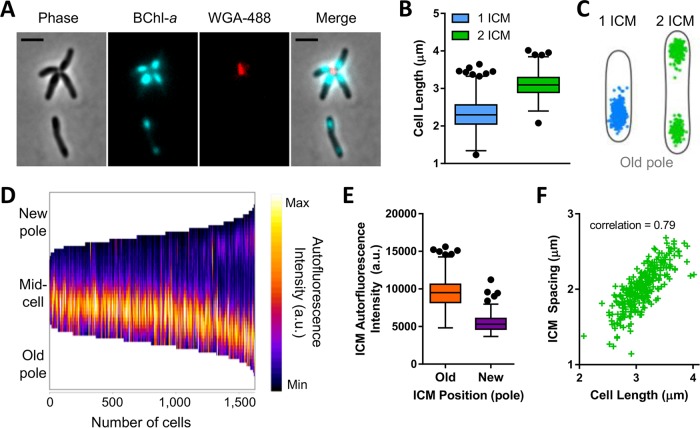
Restricted ICM localization in Rps. palustris is nonrandom. (A to F) Analysis of ICM localization in UPP-bearing WT Rps. palustris cells grown in anaerobic PMsuccYE in 8 µmol s^−1^ m^−2^ light. (A) Microscopy image of Rps. palustris cells showing phase contrast, BChl fluorescence (cyan), and UPP stained with WGA-488 (false-colored red). Scale bars, 2 µm. (B) Lengths of cells containing one or two ICMs. For 1 ICM, *n =* 1,330 cells; for 2 ICMs, *n =* 293 cells. Symbols indicate outliers (Tukey’s method). (C) Cellular position maps of BChl fluorescence centroids for ICMs detected in cells in panel B. Cell outlines depict average cell shape for each population, generated by MicrobeJ. (D) Demograph of BChl fluorescence intensities measured along the medial cell axis of all cells in panel B. Cells were sorted from shortest to longest. (E) Background-corrected mean BChl fluorescence intensity of ICMs located proximal (old) or distal (new) to the UPP-bearing pole in cells with 2 ICMs. *n =* 586 ICM maxima. Symbols indicate outliers (Tukey’s method). (F) Longitudinal distance (ICM spacing) between ICM centroids within cells containing two ICMs plotted as a function of cell length. *n =* 293 ICM pairs.

The BChl fluorescence observed in Rps. palustris was strikingly focal in nature; specifically, ICMs were localized to the slightly wider, ovoid regions near cell poles (Fig. 2C and D and Fig. 3A). Our EM images, like others previously (19), corroborated this localization pattern, showing peripheral stacks of lamellar ICMs near cell poles and an absence of ICMs in the narrower, so-called “tube” region (19) of longer cells (Fig. 2A). We hereon refer to this localization pattern as longitudinal ICM restriction.

Our ability to visualize discrete ICMs prompted us to characterize the subcellular spatial distribution of ICMs across the population. As Rps. palustris cells divide asymmetrically and produce a unipolar polysaccharide adhesin (UPP) at the old cell pole (19, 36, 37), we stained UPP with a fluorescent lectin dye to empirically orient cells (Fig. 3A). While average cell widths were comparable regardless of ICM number (data not shown), cells containing two ICMs were typically longer than cells containing a single ICM (Fig. 3B). Single ICMs were always located proximal to the UPP-bearing old pole, while in cells with two ICMs, the ICMs were positioned toward the two cell poles (Fig. 3C and D). Regardless of ICM number, the position of the UPP-proximal ICM from the UPP-bearing pole was conserved in all cells (Fig. 3C and D). A similar distance was maintained between the second ICM and the non-UPP-bearing pole in cells with two ICMs (Fig. 3C and D), although the BChl fluorescence intensity of the second ICM was consistently weaker than that of the first (Fig. 3D and E). As a consequence of the conserved ICM position relative to each pole, the distance between ICM pairs within single cells (ICM spacing) increased as a function of cell length (Fig. 3F). UPP is only present on a subset of cells in the population, but analysis of non-UPP-bearing WT cells (for which no polarity was assigned in the analysis) confirmed that the longitudinally restricted pattern of ICM localization was conserved across the population (see Fig. S3 in the supplemental material). Longitudinally restricted ICM localization was also observed in environmental Rps. palustris isolates (see Fig. S4 in the supplemental material) (38) and thus appears to be a conserved feature of this species. Additionally, longitudinal ICM restriction was maintained regardless of light availability (see Fig. S5 in the supplemental material), despite higher BChl levels and BChl fluorescence intensity in cells grown at lower light intensities (Fig. 2D). As such, we infer that ICM expansion necessary for accommodation of additional photosynthetic machinery occurs within resolute spatial constraints in Rps. palustris.

10.1128/mBio.00780-18.3 FIG S3  ICM localization pattern is conserved in non-UPP-bearing WT Rps. palustris cells. (A) Lengths of cells containing one or two ICMs. For 1 ICM, *n =* 6,493 cells; for 2 ICMs, *n =* 648 cells. (B) Demograph of BChl fluorescence intensities measured along the medial cell axis of all cells in panel A. Cells were sorted from shortest to longest. No cell polarity was assigned. (C) Longitudinal distance (ICM spacing) between ICMs within cells containing two ICMs plotted as a function of cell length. *n =* 648 ICM pairs. Download FIG S3, TIF file, 0.9 MB. Copyright © 2018 LaSarre et al. 2018 LaSarre et al. This content is distributed under the terms of the Creative Commons Attribution 4.0 International license.

10.1128/mBio.00780-18.4 FIG S4  Restricted ICM localization is conserved among diverse ecotypes of Rps. palustris. Shown are microscopy images of environmental Rps. palustris isolates grown in anaerobic PMsuccYE in 8 µmol s^−1^ m^−2^ light. Image contrast and brightness are not equivalent between fluorescence panels. Download FIG S4, TIF file, 0.8 MB. Copyright © 2018 LaSarre et al. 2018 LaSarre et al. This content is distributed under the terms of the Creative Commons Attribution 4.0 International license.

10.1128/mBio.00780-18.5 FIG S5  ICM localization is conserved across a range of light intensities. Shown are microscopy images of Rps. palustris CGA009 grown in anaerobic PMsuccYE at different light intensities. Images are from among those used for analysis in Fig. 1E. Image contrast and brightness are not equivalent between fluorescence panels. Download FIG S5, TIF file, 0.7 MB. Copyright © 2018 LaSarre et al. 2018 LaSarre et al. This content is distributed under the terms of the Creative Commons Attribution 4.0 International license.

**FIG 4:**
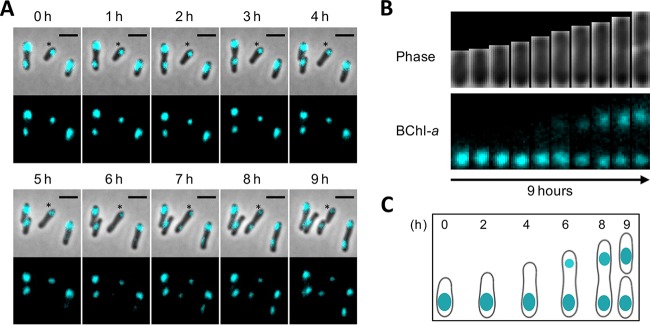
Time-lapse imaging of ICMs in Rps. palustris. (A) Time-lapse merge (upper) and BChl fluorescence (lower) montage of Rps. palustris CGA009. The time elapsed is indicated above each frame. Scale bars, 2 µm. Asterisks indicate a single cell depicted in subsequent panels. (B) Phase-contrast and BChl fluorescence profiles of the single cell from panel A over time, generated by MicrobeJ. Image contrast and brightness are not equivalent between fluorescence panels. (C) Schematic of cell shape (generated by MicrobeJ) and associated ICMs at indicated time points for the cell in panel B.

The observed correlations between cell length, ICM position, and ICM number suggested coordination of ICMs with the progression of cell growth and division. Rps. palustris grows polarly and divides by budding (19). We hypothesized that single ICMs were those of mother cells and would be retained by the mother cells upon cell division, while second ICMs developed near the new pole in predivisional cells and would be inherited by nascent daughter cells. To test this hypothesis, we monitored ICM development and position in growing Rps. palustris cells by time-lapse microscopy. Indeed, mother cells contained a single ICM, elongated from the opposite (new) pole, developed a second ICM of initially weaker BChl fluorescence near the new pole, and then completed cell division between the two ICMs (Fig. 4). Consequently, the mother and daughter cells each contained a single ICM following cell division.

### ICM localization and ICM architecture are not strictly interdependent, and neither trait fully correlates with species phylogeny.

**FIG 5:**
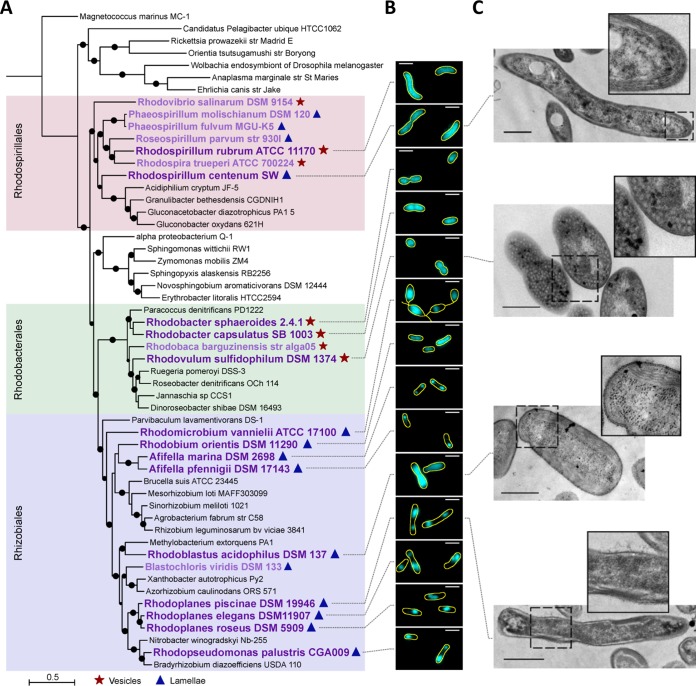
Phylogenetic analysis, BChl fluorescence patterns, and electron microscopy (EM) of select PNSB. (A) Maximum likelihood tree of non-PNSB (black text) and PNSB species (purple text) generated using a concatenated alignment of 36 housekeeping genes. M. marinus is included as an outgroup. Bootstrap values above 70% are indicated with circles. Historically noted ICM architectures are indicated by symbols (see the symbol key at the bottom of the panel). (B) BChl fluorescence (cyan) of select PNSB (large, dark purple text in panel A) with overlaid cell outlines (yellow, generated by Fiji). Scale bars, 2 µm. Fluorescence contrast and brightness are not equivalent between images. Phase-contrast, BChl fluorescence, and merged images are available in Fig. S6. (C) EM images of (from top to bottom) Rsp. centenum, Rvu. sulphidophilum, Rbl. acidophilus, and Rpl. piscinae, with accompanying enlargement of the region indicated by the dashed box for each image. Scale bars, 500 nm.

We next sought to determine if the pattern of longitudinal ICM restriction observed in Rps. palustris was specific to that species or was characteristic of the lamellar architecture. We obtained 13 additional BChl type *a* (BChl-*a*)-containing PNSB species that utilize either lamellar ICMs, similar to Rps. palustris, or vesicular ICMs, an architecture that has been most studied in other PNSB (Fig. 5A; see Table S1 in the supplemental material), and assessed ICM localization using BChl fluorescence (Fig. 5B; see Fig. S6 in the supplemental material). To gain insight into the basis of ICM diversity, we mapped ICM phenotypes (localization and/or architecture) onto a phylogenetic tree that incorporated the 21 PNSB for which genome sequences are available, including six we sequenced for this study, and for which the ICM architecture has been documented (Fig. 4; Table S1).

10.1128/mBio.00780-18.6 FIG S6  Fluorescence microscopy images of PNSB, with phase-contrast and overlay panels included. All species were grown phototrophically in 8 µmol s^−1^ m^−2^ light. Image contrast and brightness are not equivalent between fluorescence panels. Download FIG S6, TIF file, 2.2 MB. Copyright © 2018 LaSarre et al. 2018 LaSarre et al. This content is distributed under the terms of the Creative Commons Attribution 4.0 International license.

10.1128/mBio.00780-18.8 TABLE S1  Characteristics of select purple nonsulfur bacteria. Download TABLE S1, DOCX file, 0.1 MB. Copyright © 2018 LaSarre et al. 2018 LaSarre et al. This content is distributed under the terms of the Creative Commons Attribution 4.0 International license.

Of the 13 additional species examined by microscopy, seven (Rhodospirillum rubrum, Rhodospirillum centenum, Rhodobacter sphaeroides, Rhodobacter capsulatus, Rhodovulum sulfidophilum, Rhodobium orientis, and Rhodoblastus acidophilus) exhibited relatively uniform BChl fluorescence that spanned most or all of the cell, independent of cell size (Fig. 5B); we classify this pattern as nonrestricted ICM localization. Nonrestricted ICM localization was observed for both ICM architectures: while four species utilized vesicular ICMs, the other three utilized lamellar ICMs (Fig. 5A). EM imaging of three of these species illustrated that both architectures indeed spanned the length of the cells, with ICM vesicles throughout the cytoplasm of Rvu. sulfidophilum and ICM lamellae running along the cytoplasmic periphery of Rsp. centenum and Rbl. acidophilus cells (Fig. 5C).

**FIG 6:**
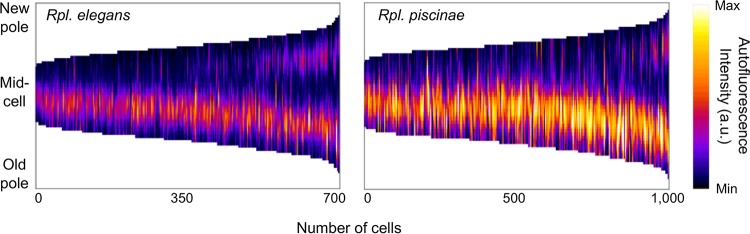
Longitudinally restricted ICM localization in Rpl. elegans and Rpl. piscinae cells. Shown are demographs of BChl fluorescence intensities measured along the medial cell axis of Rpl. elegans and Rpl. piscinae cells. Cells were sorted from shortest to longest, and polarity was determined by fluorescent lectin staining of the unipolar polysaccharide adhesin at the old cell pole. For Rpl. elegans, *n =* 714 cells; for Rpl. piscinae, *n =* 1,001 cells.

The six remaining species examined by microscopy all utilized lamellar ICMs and exhibited restricted ICM localization. Five species (Afifella marina, Afifella pfennigii, Rhodoplanes piscinae, Rhodoplanes elegans, and Rhodoplanes roseus) exhibited BChl fluorescence patterns resembling that observed in Rps. palustris (Fig. 5B). Lending support to this similarity, EM imaging of Rpl. piscinae showed stacks of peripheral ICM lamellae localized to discrete regions near the cell poles (Fig. 5C). Analysis of *Rhodoplanes* BChl fluorescence indicated that the pattern of ICM restriction was indistinguishable from that of Rps. palustris: ICM number and spacing correlated with cell length, single ICMs were located near the adhesin-producing old pole, and second ICMs maintained a similar distance from the new pole (Fig. 6; see Fig. S7 in the supplemental material). We conclude that the same pattern of longitudinal ICM restriction is shared between members of the *Rhodopseudomonas* and *Rhodoplanes* genera, and likely *Afifella* as well. In the sixth species, Rhodomicrobium vannielii, BChl fluorescence varied in both shape and uniformity of intensity between cells but localized away from the region of the cell body from which reproductive filaments emerged (Fig. 5B). Prior EM studies of Rmi. vannielii showed irregular ICM density and shape within cell bodies and the absence of ICMs and photosynthetic pigments within filaments (6, 39, 40), consistent with the BChl fluorescence observed herein. Given that ICMs are limited to the cell body and are not continuous between adjoined Rmi. vannielii cells, we have chosen to classify Rmi. vannielii as having restricted ICM localization, along with the *Afifella* and *Rhodoplanes* species.

10.1128/mBio.00780-18.7 FIG S7  Quantitative analysis of ICM localization in adhesin-bearing Rpl. elegans and Rpl. piscinae cells. (A) Microscopy image of cells showing BChl fluorescence (cyan) and polar adhesin stained with ConA-488 (false-colored red). Scale bars, 2 µm. Image contrast and brightness are not equivalent between fluorescence panels. (B) Lengths of cells containing one or two ICMs. For Rpl. elegans, for 1 ICM, *n =* 496 cells, and for 2 ICMs, *n =* 218 cells; for Rpl. piscinae, for 1 ICM, *n =* 846 cells, and for 2 ICMs, *n =* 155 cells. (C) Longitudinal distance (ICM spacing) between ICMs within cells containing two ICMs plotted as a function of cell length. For Rpl. elegans, *n =* 218 ICM pairs; for Rpl. piscinae, *n =* 155 ICM pairs. Download FIG S7, TIF file, 0.5 MB. Copyright © 2018 LaSarre et al. 2018 LaSarre et al. This content is distributed under the terms of the Creative Commons Attribution 4.0 International license.

While neither ICM localization nor architecture strictly correlated with phylogeny (Fig. 5), we were able to draw several inferences about the evolutionary history of ICM patterning. All members of *Rhodospirillales* and *Rhodobacterales* exhibited nonrestricted localization, while *Rhizobiales* members exhibited both restricted and nonrestricted localization. We therefore infer an ancestral state of nonrestricted ICM patterning, with restricted localization arising as a late innovation in the *Rhizobiales* after their split from the *Rhodobacterales* (Fig. 5). Given that restricted localization appears in only seven of the nine *Rhizobiales* species studied here (Rbi. orientis and Rbl. acidophilus appear to have derived, nonrestricted ICM patterns), while the lamellar architecture has been maintained across the order, we further infer that the ancestral PNSB *Rhizobiales* species had restricted lamellar ICMs. Separately, we infer that the ancestral PNSB *Rhodobacterales* species had nonrestricted vesicular ICMs, as these traits are conserved across the order. With regard to the broader evolutionary history of ICM architecture, *Rhodobacterales* and *Rhizobiales* each utilized a single architecture (vesicles and lamellae, respectively), while *Rhodospirillales* contained members with each architecture type, and these types did not cluster as separate lineages. The presence of both architectures within *Rhodospirillales* confounds any inference of the ancestral PNSB ICM architecture. Overall, we conclude that ICMs are subject to differential spatial organizations among PNSB but not in strict accordance with ICM architecture or species phylogeny.

## DISCUSSION

Here we revealed diversity in ICM localization among PNSB by exploiting the natural autofluorescence of BChl. Because BChl is a native component of the ICM-residing photosynthetic machinery, this method did not require genetic manipulation (i.e., fluorescent protein fusions), thereby enabling characterization of diverse PNSB, many for which genetic tools are unavailable and in which fluorescent tags are not generally amenable (due to anaerobic and illuminated growth conditions preventing proper maturation of fluorescent proteins and causing photobleaching, respectively). However, the BChl fluorescence approach has some limitations. BChl fluorescence intensity correlates with cellular BChl content (Fig. 2E); consequently, PNSB with prohibitively low cellular BChl content will not be amenable to this approach, as an absence of BChl fluorescence could result from low BChl and/or ICM abundance rather than the complete absence of ICMs. Thus, lack of BChl fluorescence should be interpreted carefully and preferably corroborated using additional techniques such as EM, as was done here. Relatedly, while the BChl fluorescence pattern indicates ICM localization with regard to the cell long axis, it does not inform on ICM architecture. For example, nonrestricted BChl fluorescence was derived from both ICM lamellae and ICM vesicles (Fig. 5), and while the restricted localization observed herein was specific to lamellar ICMs in a subset of *Rhizobiales* members, it is possible that examination of additional species would identify non-*Rhizobiales* and/or vesicle-bearing PNSB with restricted ICM localization.

We were intrigued by the diversity of ICM localization in the examined PNSB, specifically the differential use of perceived available cell space. Many species elaborated ICMs along the entire length of the cell, but other species utilized only patches of the cytoplasmic periphery for ICMs (Fig. 5B), despite that all ICMs presumably serve to enhance light capture and energy transformation (5, 9, 10). Our results therefore indicate that ICM function does not necessitate maximizing surface area (i.e., using all available cell space). An ensuing question, then, is whether different localization patterns impart physiological benefits. Perhaps ICM localization diversity reflects differences in the photosynthetic capacity of ICMs and/or the cellular energy demands among PNSB, wherein certain species can transform sufficient energy using a smaller portion of their cell space and thereby reduce the metabolic costs of ICM synthesis. While all PNSB utilize a core photosynthetic machinery that consists of a reaction center intimately associated with light-harvesting complexes (i.e., light-harvesting complex 1 [LH1]), PNSB differ in the number and/or type of peripheral light-harvesting complexes (LH2) used for light capture (41, 42). Moreover, some species can alter both the amount and type of LH2 present in response to light availability (7, 10, 43, 44). This diversity in LH2 utilization could underlie inherent differences in photosynthetic capacity between species. However, LH2 inventory itself does not easily account for ICM localization—nor ICM architecture for that matter. PNSB that lack LH2 can have either longitudinally restricted or nonrestricted localization and either lamellar or vesicular ICMs (for example, *Afifella* spp. and Rsp. rubrum) (45, 46). The same is true for PNSB that contain multiple copies of LH2 complex genes (for example, Rps. palustris, Rbl. acidophilus, and Rba. sphaeroides) (43, 44, 47, 48). Further work will be necessary to resolve if a relationship exists between ICM localization and LH2 diversity and/or energy transformation. A nonexclusive possibility is that ICM localization diversity reflects evolution amid other facets of cell physiology. For example, ICM localization might need to be compatible with the mode of cell growth and division in a given species. More specifically, perhaps the restricted ICM localization in Rps. palustris and *Rhodoplanes* species evolved because these species cannot complete cell division across ICMs; longitudinal restriction could thereby function to keep the ICM away from the future division site. While there was no strict correlation between ICM architecture or localization and mode of cell division (binary fission or budding) in the examined PNSB (Table S1), there is currently a limited understanding of molecular determinants and mechanisms distinguishing different modes of bacterial growth and division. It is possible that the general classification of “budding” actually incorporates subclasses of budding that differ on the molecular level; restricted ICM localization could correlate with a specific budding subclass. We anticipate that future insights into bacterial reproduction mechanisms will help clarify any connections between ICMs and cell growth and division.

Regardless of division mode, both nonrestricted and longitudinally restricted ICM localization would be expected to promote ICM inheritance, thereby ensuring that the metabolic (in this case, photosynthetic) capacity of cells is maintained across the cell cycle, as is true for protein-encapsulated, CO_2_-fixing carboxysome compartments (49). However, ICMs are conditionally made in PNSB (22, 26, 28), and ICM localization patterns could differentially impact population fitness during shifts between phototrophic and nonphototrophic (e.g., aerobic) conditions. For example, longitudinally restricted ICM localization could increase population heterogeneity upon phototrophic-aerobic shift, as mother cells would be expected to retain ICMs while generating ICM-lacking daughter cells. In contrast, PNSB with unrestricted ICMs would be expected to exhibit progressive dilution of ICMs in both mother and daughter cells through rounds of cell division. Additional studies are needed to interrogate the impact of ICM inheritance on cell fitness under both constant phototrophic conditions and upon shifts to aerobic conditions.

Apart from potential benefits conferred by ICM localization, another fundamental question remains: how is ICM localization established and maintained? Presumably, ICM localization is the collective result of the site(s) of ICM synthesis and the extent to which ICMs can expand. One intriguing possibility is that sites of ICM synthesis could be conserved among PNSB despite the fact that ICMs expand to occupy different portions of the cells. In this way, nonrestricted ICM localization may be fundamentally systematic in nature, similar to that of longitudinally restricted ICMs, although visually unapparent. In Rba. sphaeroides, ICM vesicles develop from curved sites in the CM (8); little is known about the sites of formation for vesicular or lamellar ICMs in other PNSB. Molecular determinants that influence ICM localization are currently unknown but could include cytoskeletal-like proteins, which are known to coordinate the spatial arrangement of two other bacterial compartments, carboxysomes (49) and the membrane-bound, magnetic field-sensing magnetosomes (50, 51). PNSB within *Rhizobiales* lack the actin homologue MreB, but all PNSB have predicted cytoskeletal-like ParA proteins that could be involved in ICM localization. PNSB might also harbor unpredicted cytoskeletal-like proteins, as these proteins are highly divergent and are thus difficult to identify from sequence alone (52). The diversity in ICM localization revealed herein (Fig. 5) suggests that different synthesis and/or restriction mechanisms are at play, even for architecturally similar ICMs. Identification of molecular factors governing ICM localization will be invaluable to understand the biological relevance of distinct localization patterns and will foster a broader perspective on subcellular organization in bacteria.

## MATERIALS AND METHODS

### Strains, plasmids, and growth conditions.

All species, strains, and plasmids used in this study are listed in Table S2 in the supplemental material. Rhodobacter sphaeroides 2.4.1 and Rhodospirillum rubrum ATCC 11170 were provided courtesy of Gary Roberts (University of Wisconsin, Madison). Rhodospirillum centenum SW and Rhodobacter capsulatus SB 1003 were provided courtesy of Carl Bauer (Indiana University). Rhodoblastus acidophilus DSM 137 was provided courtesy of Michael Madigan (Southern Illinois University). Seven strains were purchased from DSMZ (Deutsche Sammlung von Mikroorganismen und Zellkulturen GmbH, Braunschweig, Germany): Afifella marina DSM 2698, Afifella pfennigii DSM 17143, Rhodobium orientis DSM 11290, Rhodoplanes elegans DSM 11970, Rhodoplanes piscinae DSM 19946, Rhodoplanes roseus DSM 5909, and Rhodovulum sulfidophilum DSM 1374.

10.1128/mBio.00780-18.9 TABLE S2  Bacterial strains, plasmids, and primers used in this study. Download TABLE S2, DOCX file, 0.1 MB. Copyright © 2018 LaSarre et al. 2018 LaSarre et al. This content is distributed under the terms of the Creative Commons Attribution 4.0 International license.

PNSB were grown photoheterotrophically in the following media. Rhodopseudomonas palustris, Rba. sphaeroides, Rsp. rubrum, and Rhodomicrobium vannielii were cultivated in defined mineral (PM) medium (53) supplemented with 5 mM succinate and 0.1% (wt/vol) yeast extract (YE) (PMsuccYE). Rsp. centenum was cultivated on CENS medium (54). Rba. capsulatus was cultivated in modRM2 medium (RM2 [55] modified by substitution with the trace elements solution described in reference 56 in place of trace elements solution 8) supplemented with 5 mM succinate and 0.1% (wt/vol) YE. All three *Rhodoplanes* species were cultivated in modRM2 supplemented with 20 mM pyruvate and 0.1% (wt/vol) YE. Marine species were cultivated in modRM2 supplemented with 20 mM pyruvate and 0.1% (wt/vol) YE plus either 2% (wt/vol) NaCl (*Afifella* species and Rvu. sulfidophilum) or 5% (wt/vol) NaCl (Rbi. orientis). Rbl. acidophilus was cultivated in modABM (acidic basal medium [57] modified by substitution with the trace elements solution from reference 56 in place of the trace elements solution of Pfennig and Lippert) supplemented with 20 mM succinate. For photoheterotrophic cultures, media were made anaerobic by bubbling with Ar and then sealing with rubber stoppers and screw caps. Cultures were incubated statically at 30°C at discrete distances from 60-W (750-lm) soft white halogen bulbs to achieve the light intensities described in the text. Anaerobic, N_2_O-respiring Rps. palustris cultures were cultivated in sealed tubes containing anaerobic PM supplemented with 10 mM butyrate and 40 µM NaNO_3_ (necessary for induction of *nos* genes required for N_2_O reduction) (unpublished) and flushed with N_2_O prior to static incubation at 30°C in darkness. Aerobic Rps. palustris cultures were grown in 20 ml PMsuccYE in 125-ml flasks at 30°C in darkness with shaking at 225 rpm.

### Generation of Rps. palustris mutants.

The *crtI* deletion vector (pJQcrtIKO) was generated by PCR amplifying flanking DNA regions using primer pairs BL530/BL531 and BL532/BL533 (Table S2). The two products were fused by *in vitro* ligation at their engineered SacI sites. The fusion was subsequently amplified by PCR using primers BL530/BL533 and then ligated into the XbaI site of pJQ200SK. The *bchXYZ* deletion vector (pJQbchXYZKO) was generated by PCR amplification of flanking DNA regions using primer pairs BL557/BL558 and BL559/BL560 (Table S2). These primers were designed using the NEBuilder tool (New England Biolabs) for isothermal assembly into plasmid pJQ200SK. PCR products were mixed with XbaI-digested pJQ200SK, assembled using Gibson Assembly (NEB), and transformed into NEB10β Escherichia coli cells (New England Biolabs) cultivated on Luria-Bertani medium (Difco).

Deletion vectors were introduced into Rps. palustris by electroporation (58). Mutants were generated using sequential selection and screening as described previously (59). Mutant genotypes were confirmed by PCR and sequencing. When necessary, gentamicin was included at 15 µg/ml for E. coli or 100 µg/ml for Rps. palustris.

### Analytic procedures.

Culture growth was monitored by optical density at 660 nm (OD_660_) using a Genesys 20 visible spectrophotometer (Thermo-Fisher). Room temperature absorbance spectra of whole cells resuspended in 60% (wt/vol) sucrose in PM were recorded using a Synergy MX spectrofluorimeter (BioTek). Light intensity was monitored using a LI-250A light meter equipped with a LI-190R quantum sensor (LI-COR).

Bacteriochlorophyll *a* (BChl-*a*) was extracted and quantified as previously described (28, 60). Briefly, cells were centrifuged and resuspended in 600 µl phosphate-buffered saline (PBS), at which point sample OD_660_ was recorded. Cells were then pelleted again, resuspended in 20 µl H_2_O, mixed with 1 ml 7:2 (vol/vol) acetone-methanol solvent, and incubated at room temperature in darkness for 90 min. Cell debris was removed by centrifugation (at maximum speed for 5 min), and extracted BChl-*a* was detected by recording supernatant absorbance at 770 nm (*A*_770_). BChl-*a* content is reported normalized to sample optical density (*A*_770_/OD_660_).

### Staining polar adhesins with fluorescent lectins.

Rps. palustris or *Rhodoplanes* cells were resuspended in PBS and incubated with Alexa Fluor 488-conjugated wheat-germ agglutinin (WGA-488) or concanavalin A (ConA) (Invitrogen), respectively, at a final concentration of 0.5 µg/ml for 5 min prior to fluorescence microscopy imaging.

### Fluorescence microscopy.

General cell imaging was performed on 1.5% (wt/vol) agarose pads made with unsupplemented media. For time-lapse microscopy, log-phase Rps. palustris cells from photoheterotrophic cultures grown in 8 µmol s^−1^ m^−2^ light were applied to 1% (wt/vol) agarose pads made with PMsuccYE and sealed with Valap (1:1:1 Vaseline [petrolatum]-lanolin-paraffin) prior to imaging. Slides were left incubating on the microscope at room temperature between captures. All imaging was performed on a Nikon Eclipse E800 equipped with a 100× Plan Apo Ph3 DM oil immersion objective, Xcite 120 metal halide lamp (Excelitas Technologies), 83700 DAPI-FITC-Texas Red triple-filter cube (Chroma Technologies), and a Photometrics Cascade 1K EM-CCD camera. Images were captured using NIS Elements (Nikon). As the microscope and filter cube used herein are no longer commercially available, information regarding additional BChl-*a* fluorescence-compatible microscopes and filter sets is provided in Table S3 in the supplemental material.

10.1128/mBio.00780-18.10 TABLE S3  Alternative microscopes and filter sets for visualizing BChl-*a* fluorescence. Download TABLE S3, DOCX file, 0.1 MB. Copyright © 2018 LaSarre et al. 2018 LaSarre et al. This content is distributed under the terms of the Creative Commons Attribution 4.0 International license.

### Image analysis.

All BChl fluorescence images were subject to an elastic transformation to correct a consistent spatial distortion resulting from using non-UV-corrected microscope components. A reference transformation matrix was generated from phase-contrast and epifluorescence images of a single, crowded field of Rba. sphaeroides cells (diffuse BChl fluorescence) using the bUnwarpJ (61) plug-in in Fiji (62); the resulting matrix transformation was then applied to images of all 14 species prior to analysis. Detection of cells, BChl fluorescence, and polar adhesins was performed using MicrobeJ (63). As necessary, the MicrobeJ Manual Editor was used to split touching cells. All cell shape parameters and autofluorescent foci (ICM) positions were measured by MicrobeJ. Demographs and XY cell maps were generated by MicrobeJ. Image brightness and contrast were adjusted as necessary within Fiji (62) to aid in image visualization; when visually comparing cellular fluorescence intensity between samples (Fig. 2), adjustments were kept constant across images.

### Electron microscopy.

Electron microscopy was performed at the Indiana University Electron Microscopy Center. Samples were fixed in 2.0% glutaraldehyde–1% tannic acid in unsupplemented PM medium for 1 h at room temperature, with the exception of Rvu. sulfidophilum, which was fixed in 2.0% glutaraldehyde in modRM2 medium containing 2% (wt/vol) NaCl. Fixed samples were placed on ice and put through four changes of unsupplemented medium, with incubation in 1% osmium tetroxide in 0.1 M sodium cacodylate buffer (pH 7.2) for 1 h, followed by two changes of sodium cacodylate buffer. Samples were then put through a graded ethanol dehydration series to 100% ethanol with en bloc staining of 2% uranyl acetate for 30 min at the 75% ethanol step. Samples were subsequently put through three changes of 100% ethanol at room temperature, followed by infiltration using low-viscosity embedding resin (Electron Microscopy Sciences, Hatfield, PA) with four changes of resin before the samples were polymerized at 65°C for 18 h. Ultrathin sections of 85-nm thickness on 300 mesh copper grids were obtained using a Leica Ultracut UCT ultramicrotome. Grids were stained with saturated uranyl acetate and lead citrate and imaged using a JEM-1400Plus 120-kV transmission electron microscope (JEOL United States, Inc.) with a Gatan OneView 4K by 4K camera.

### Genome sequencing.

Genome sequencing of the six bacterial strains indicated below was performed at the Indiana University Center for Genomics and Bioinformatics. Paired-end libraries generated with the NEXTflex Rapid DNA-seq kit (Bioo Scientific) were sequenced using standard Illumina sequencing protocols. Reads were adapter trimmed and quality filtered using Trimmomatic v0.33, requiring a minimum quality of q20 and a minimum read length of 50 nucleotides after trimming. Reads were assembled using the software SPAdes v3.9.1. Draft genomes were annotated using the NCBI Prokaryotic Genome Annotation Pipeline v4.2 (64).

### Phylogenetic analysis.

Whole-genome data were obtained from the genome database at the National Center for Biotechnology Information (65). Conserved housekeeping gene amino acid sequences were automatically identified, aligned, and concatenated using Phylosift (66). Maximum likelihood phylogeny reconstruction and bootstrap support estimation were performed in RAxML v8.2.9 (67), and trees were visualized using iTOL v4.0.2 (68).

### Accession numbers.

The whole-genome shotgun projects have been deposited at DDBJ/ENA/GenBank under the following accession numbers: Rhodoblastus acidophilus DSM 137, NPET00000000; Rhodobium orientis DSM 11290, NPEV00000000; Rhodoplanes elegans DSM 11907, NPEU00000000; Rhodoplanes piscinae DSM 19946, NPEW00000000; Rhodoplanes roseus DSM 5909, NPEX00000000; Afifella marina DSM 2698, NPBC00000000. For each project, the version described in this paper is version XXXX01000000.
