# Supplementary figures and images for: Restricted Localization of Photosynthetic Intracytoplasmic Membranes (ICMs) in Multiple Genera of Purple Nonsulfur Bacteria
Source: mBio. 2018 Jul 3;9(4):e00780-18. doi: 10.1128/mBio.00780-18 (PMC6030561; doi:10.1128/mBio.00780-18)

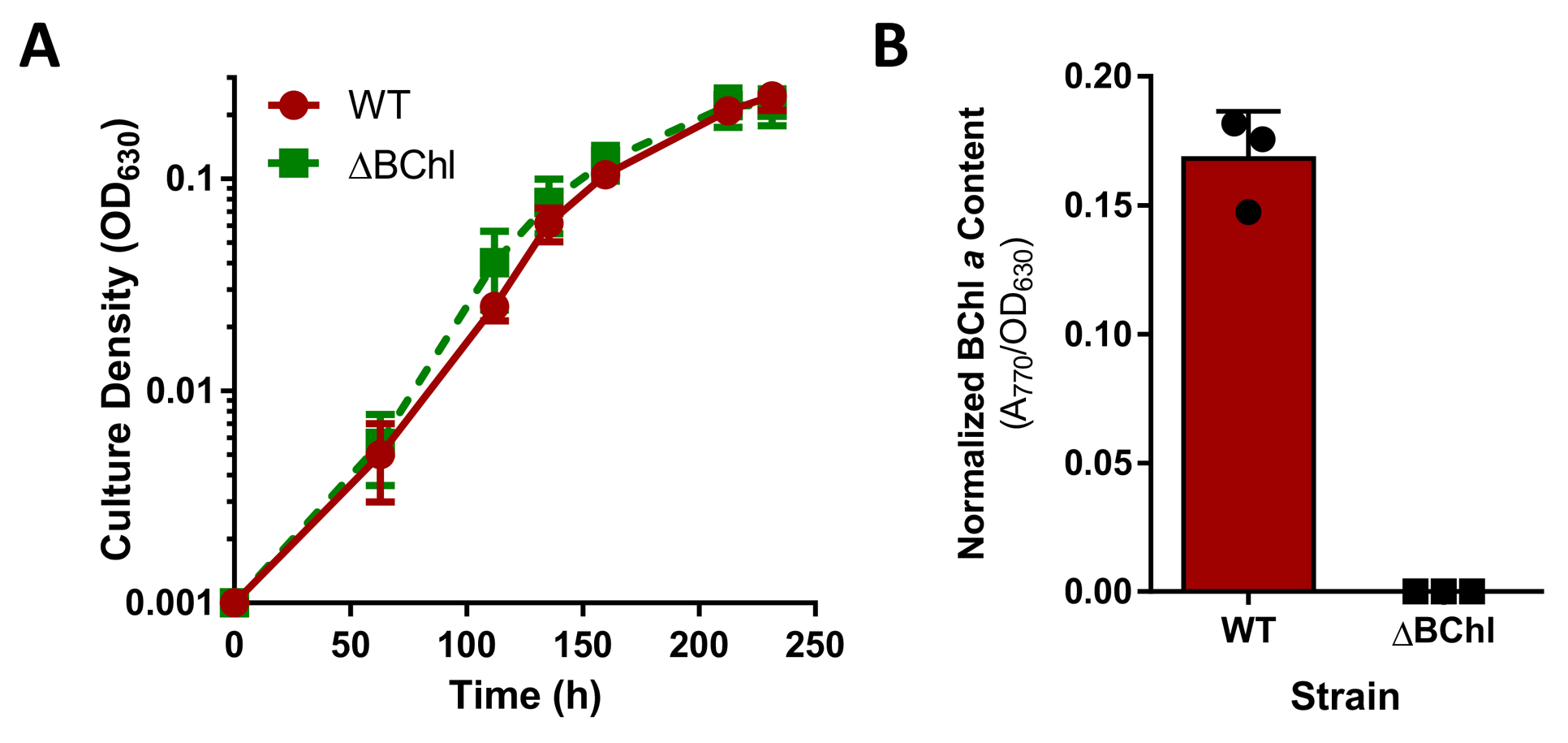

Supplement: FIG S1 [file mbo004183956sf1.tif]

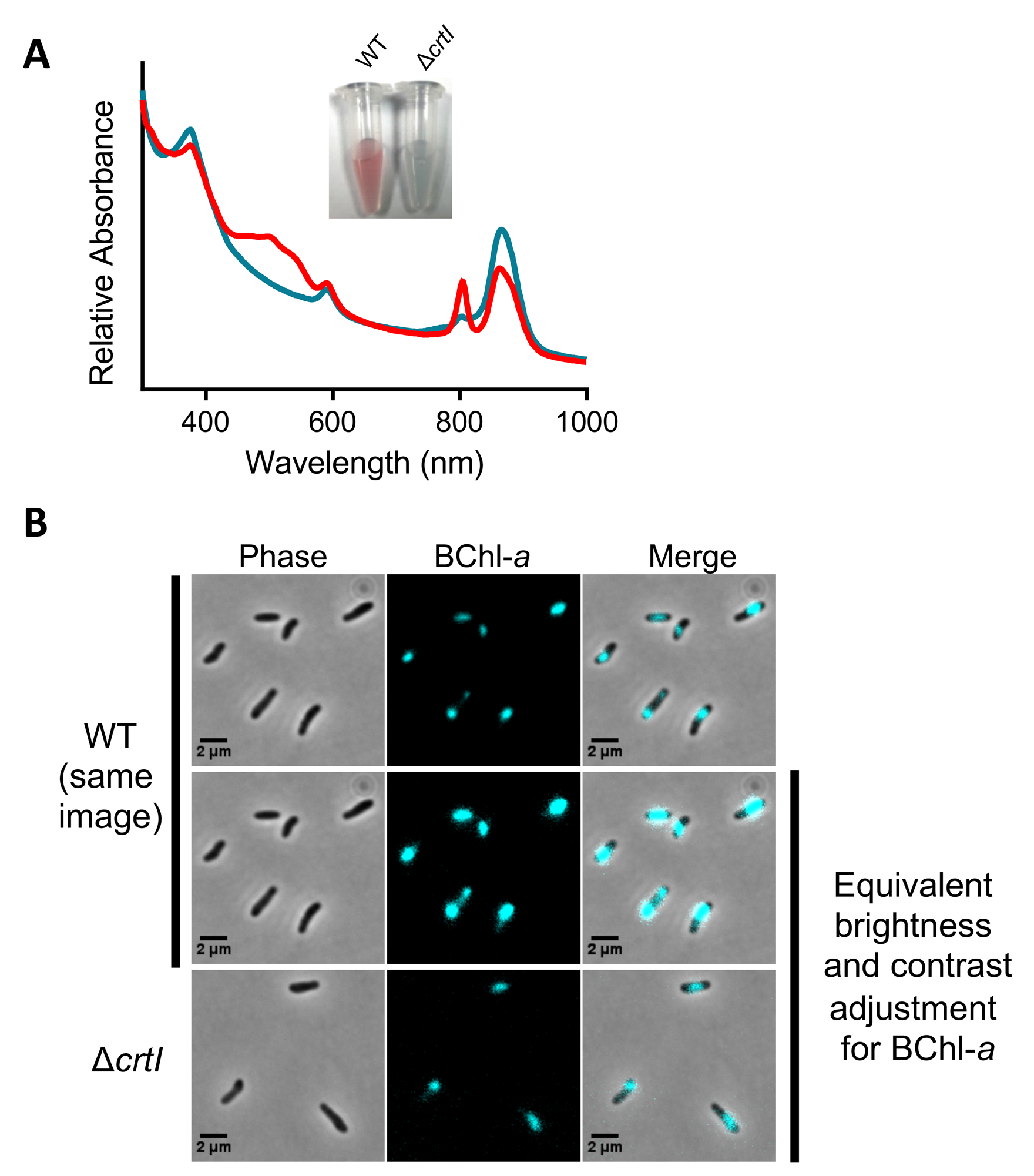

Supplement: FIG S2 [file mbo004183956sf2.tif]

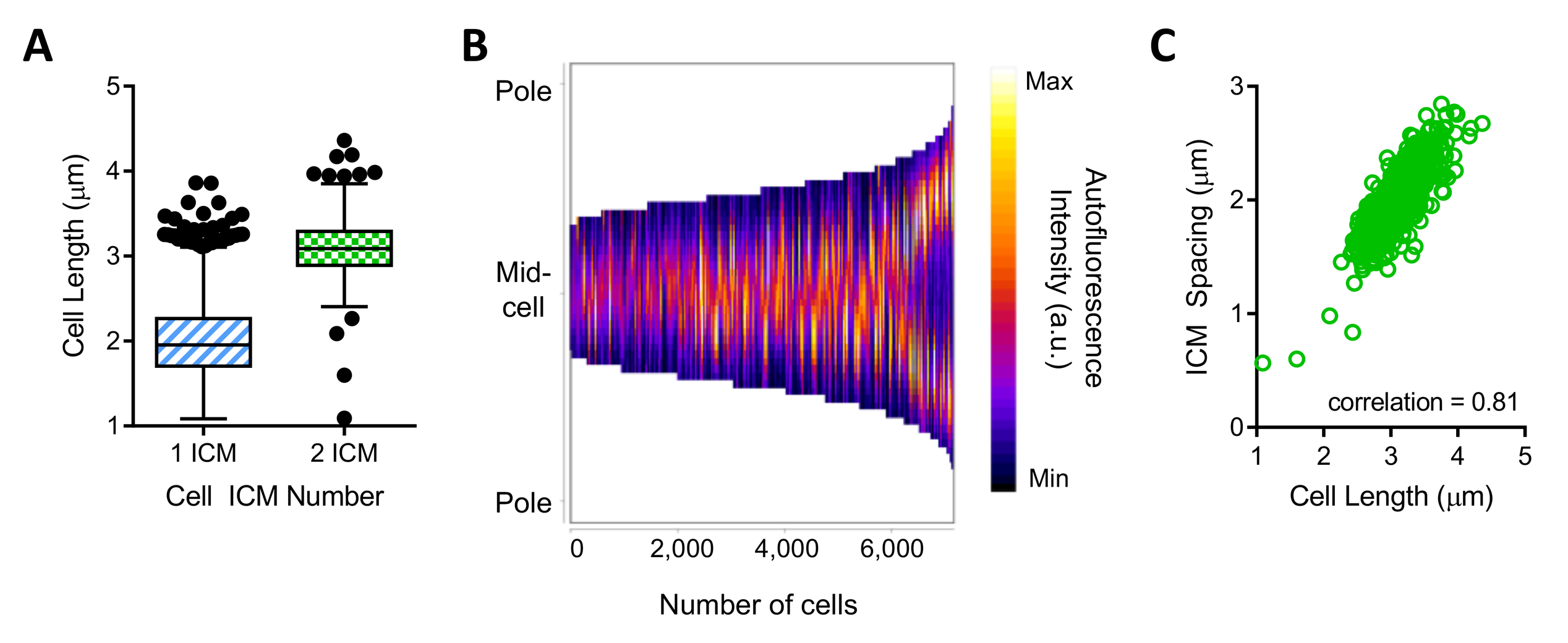

Supplement: FIG S3 [file mbo004183956sf3.tif]

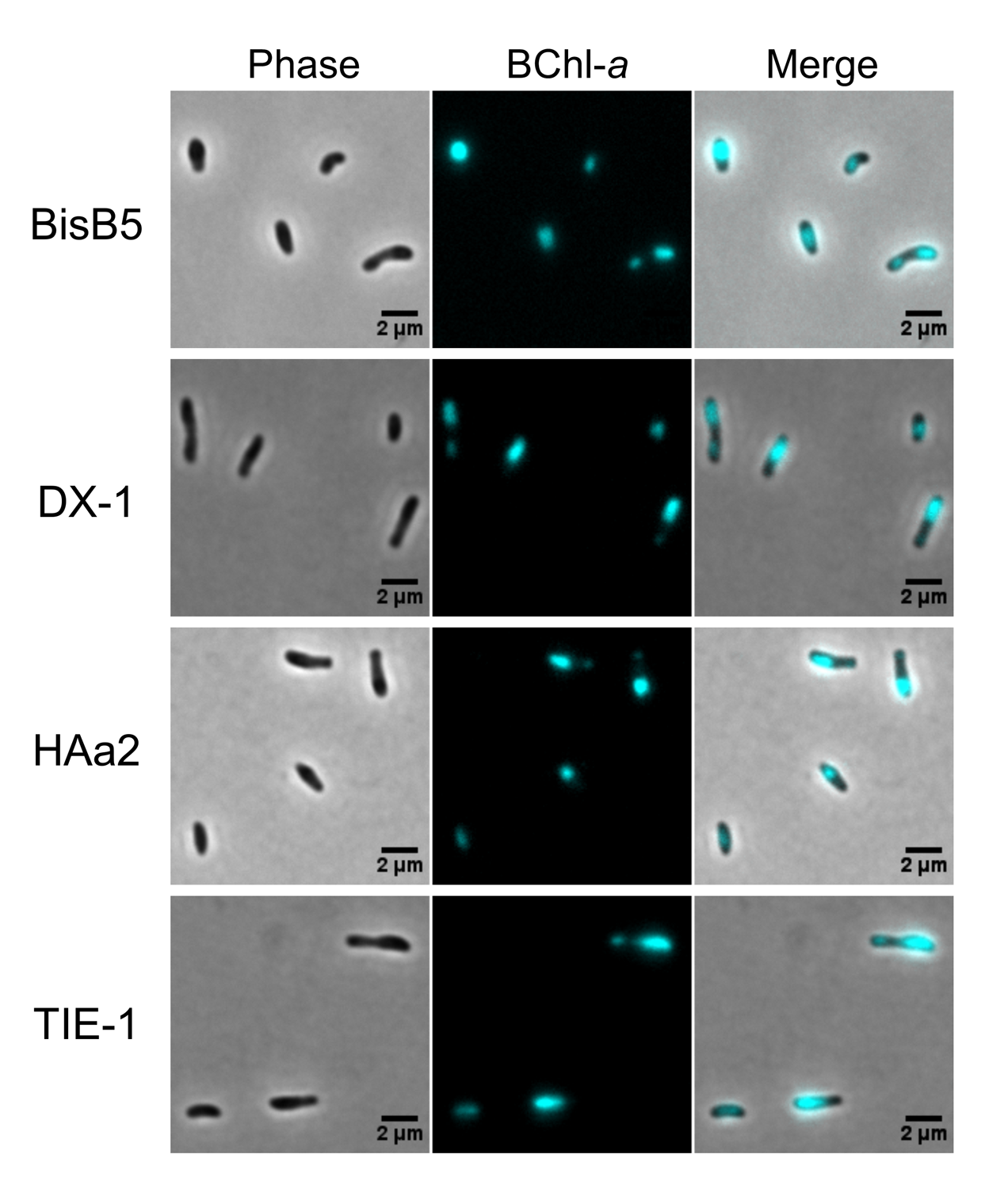

Supplement: FIG S4 [file mbo004183956sf4.tif]

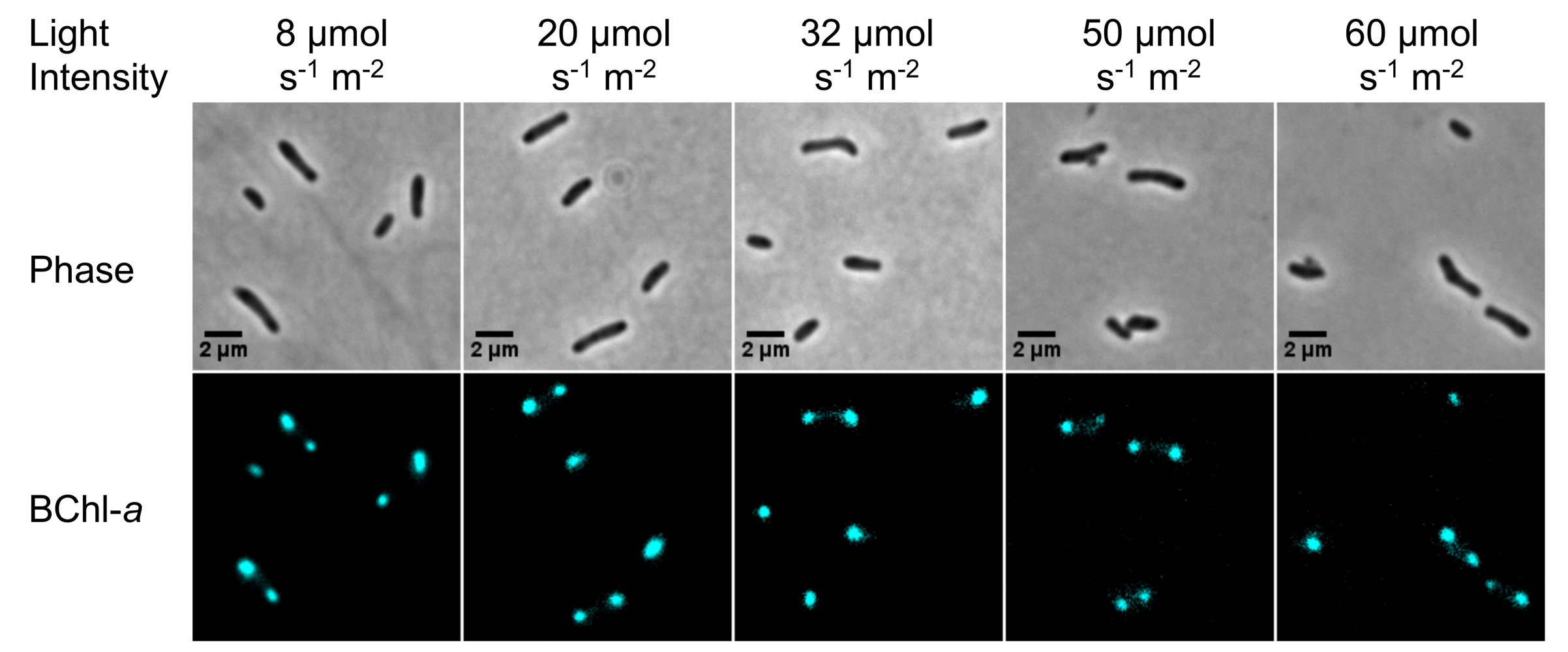

Supplement: FIG S5 [file mbo004183956sf5.tif]

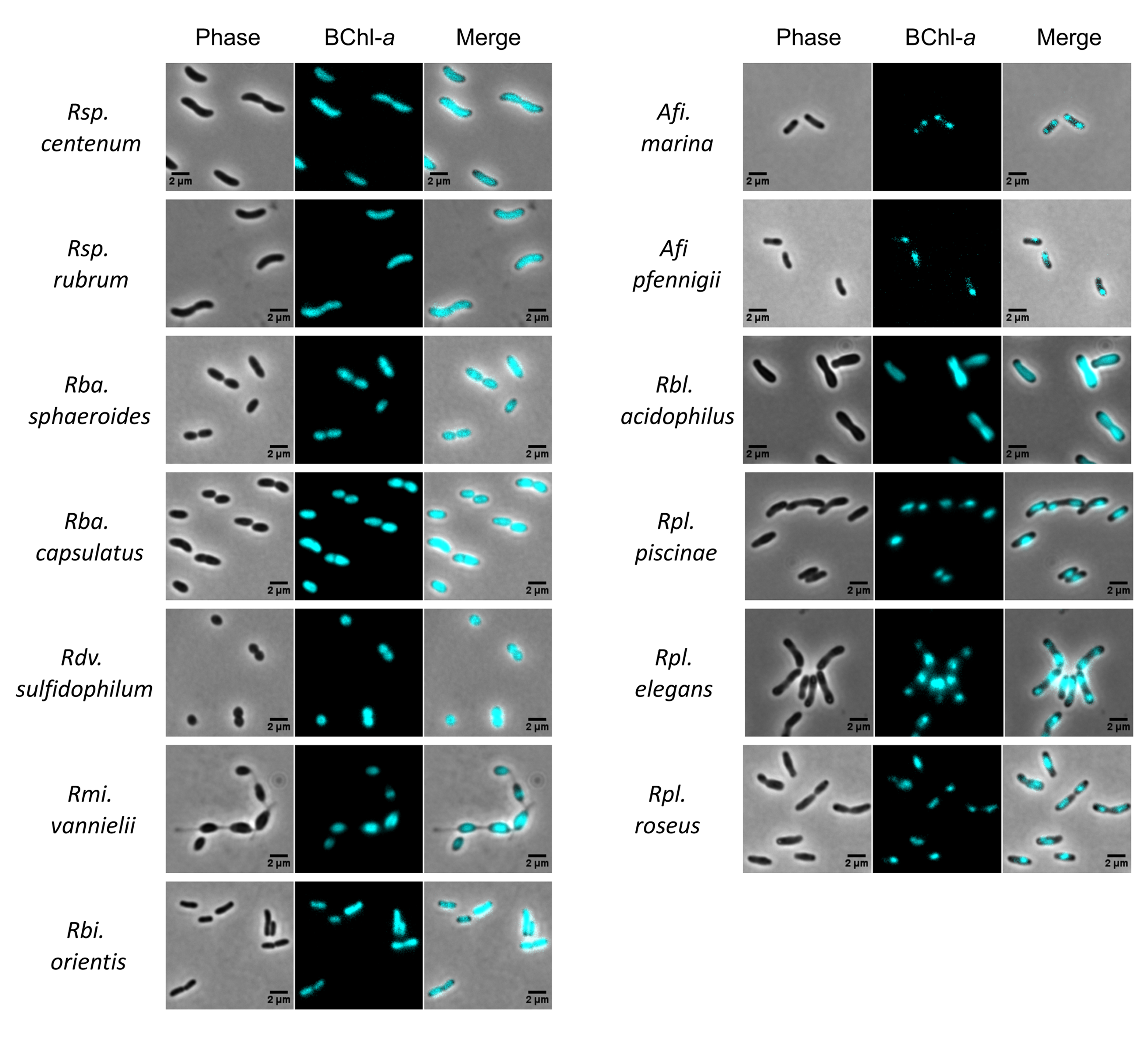

Supplement: FIG S6 [file mbo004183956sf6.tif]

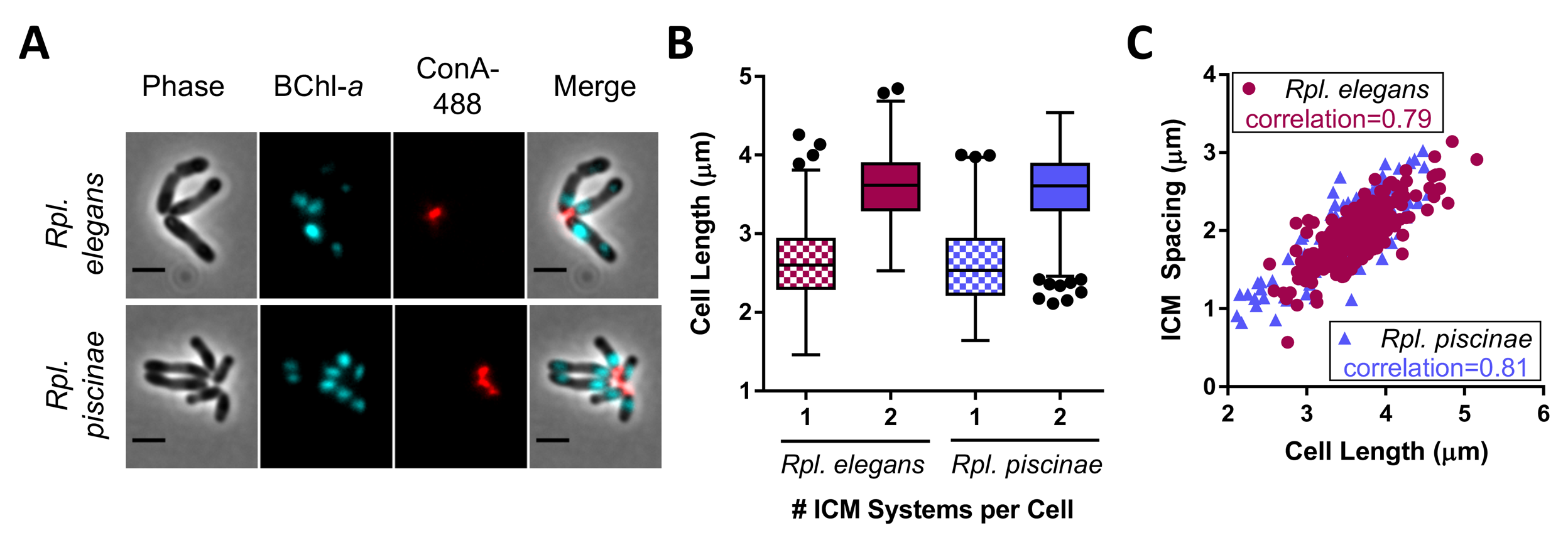

Supplement: FIG S7 [file mbo004183956sf7.tif]
