# Supplementary material for: Restricted Localization of Photosynthetic Intracytoplasmic Membranes (ICMs) in Multiple Genera of Purple Nonsulfur Bacteria
Source: mBio. 2018 Jul 3;9(4):e00780-18. doi: 10.1128/mBio.00780-18 (PMC6030561; doi:10.1128/mBio.00780-18)
Supplement: TABLE S1 [file mbo004183956st1.docx]

**Table S1. Characteristics of select purple non-sulfur bacteria.**

| **Species^a^** | **Order** | **Reproduction Type^b^** | **Rosettes^c^** | **ICM Type^d^** | **BChl Type** | **Carotenoid Series^e^** | **Habitat** | **Reference** |
| --- | --- | --- | --- | --- | --- | --- | --- | --- |
| *Rhodovibrio salinarum* | Rhodospirillales | Binary Fission | No | V | *a* | Spirilloxanthin | Saltern | (1, 2) |
| ***Rhodospirillum centenum***^*^ |  | Binary Fission | No | L | *a* | Spirilloxanthin | Freshwater | (3, 4) |
| *Phaeospirillum molischianum* |  | Binary Fission | No | L | *a* | Spirilloxanthin | Freshwater | (1, 5, 6) |
| *Phaeospirillum fulvum* |  | Binary Fission | No | L | *a* | Spirilloxanthin | Freshwater | (1, 6, 7) |
| *Roseospirillum parvum* |  | Binary Fission | No | L | *a* | Spirilloxanthin | Marine | (8) |
| ***Rhodospirillum rubrum*** |  | Binary Fission | No | V | *a* | Spirilloxanthin | Freshwater | (1, 9) |
| *Rhodospira trueperi* |  | Binary Fission | No | V | *b* | Spirilloxanthin | Marine | (10) |
| ***Rhodobacter sphaeroides*** | Rhodobacterales | Binary Fission | No | V | *a* | Spheroidene | Freshwater | (11) |
| ***Rhodobacter capsulatus*** |  | Binary Fission | No | V | *a* | Spheroidene | Freshwater | (11) |
| *Rhodobaca barguzinensis* |  | Binary Fission | No | V | *a* | Spheroidene | Marine | (12) |
| ***Rhodovulum sulfidophilum*** |  | Binary Fission | No | V | *a* | Spheroidene | Marine | (13) |
| ***Rhodomicrobium vannielii*** | Rhizobiales | Budding | No | L | *a* | Spirilloxanthin | Freshwater | (14, 15) |
| ***Rhodobium orientis*** |  | Budding | Yes | L | *a* | Spirilloxanthin | Marine | (16) |
| ***Afifella marina*** |  | Budding | No | L | *a* | Spirilloxanthin | Marine | (17, 18) |
| ***Afifella pfennigii*** |  | Budding | Yes | L | *a* | Spirilloxanthin | Marine | (18, 19) |
| ***Rhodoblastus acidophilus*** |  | Budding | Yes | L | *a* | Spirilloxanthin | Freshwater | (20, 21) |
| *Blastochloris viridis* |  | Budding | Yes | L | *b* | Spirilloxanthin | Freshwater | (22, 23) |
| ***Rhodoplanes piscinae*** |  | Budding | Yes | L | *a* | Spirilloxanthin | Freshwater | (24) |
| ***Rhodoplanes elegans*** |  | Budding | Yes | L | *a* | Spirilloxanthin | Freshwater | (25) |
| ***Rhodoplanes roseus*** |  | Budding | No | L | *a* | Spirilloxanthin | Freshwater | (25, 26) |
| ***Rhodopseudomonas palustris*** |  | Budding | Yes | L | *a* | Spirilloxanthin | Freshwater | (7, 27) |

^a^ Species in bold are those empirically examined herein

^b^ Modes of reproduction are those historically reported based on phase-contrast microscopy

^c^ Rosette formation as historically reported and/or empirically observed during the course of this work

^d^ V, vesicles; L, lamellae

^e^ Carotenoid content described in indicated references and/or (28)

^f^ Number indicates *puc* gene content in the specific strain of each species indicated on the tree in Fig 5

^*^ Species also known as *Rhodocista centenaria* (29)

**Table S1 References**

1. Imhoff JF, Petri R, Suling J. 1998. Reclassification of species of the spiral-shaped phototrophic purple non-sulfur bacteria of the alpha-Proteobacteria: description of the new genera *Phaeospirillum* gen. nov., *Rhodovibrio* gen. nov., *Rhodothalassium* gen. nov. and *Roseospira* gen. nov. as well as transfer of *Rhodospirillum fulvum* to *Phaeospirillum fulvum* comb. nov., of *Rhodospirillum molischianum* to *Phaeospirillum molischianum* comb. nov., of *Rhodospirillum salinarum* to *Rhodovibrio salexigens*. Int J Syst Bacteriol 48 Pt 3:793-8.

2. Nissen H, Dundas ID. 1984. *Rhodospirillum salinarum* sp. nov., a Halophilic Photosynthetic Bacterium Isolated from a Portuguese Saltern. Arch Microbiol 138:251-256.

3. Favinger J, Stadtwald R, Gest H. 1989. *Rhodospirillum centenum*, sp. nov., a thermotolerant cyst-forming anoxygenic photosynthetic bacterium. Antonie Van Leeuwenhoek 55:291-6.

4. Yildiz FH, Gest H, Bauer CE. 1991. Genetic analysis of photosynthesis in *Rhodospirillum centenum*. J Bacteriol 173:4163-70.

5. Giesberger G. 1947. Some observations on the culture, physiology and morphology of some brown-red *Rhodospirillum*-species. Antonie Van Leeuwenhoek 13:135-148.

6. Takaichi S, Maoka T, Sasikala C, Ramana Ch V, Shimada K. 2011. Genus specific unusual carotenoids in purple bacteria, *Phaeospirillum* and *Roseospira*: structures and biosyntheses. Curr Microbiol 63:75-80.

7. van Niel CB. 1944. The Culture, General Physiology, Morphology, and Classification of the Non-Sulfur Purple and Brown Bacteria. Bacteriol Rev 8:1-118.

8. Glaeser J, Overmann J. 1999. Selective enrichment and characterization of *Roseospirillum parvum*, gen. nov. and sp. nov., a new purple nonsulfur bacterium with unusual light absorption properties. Arch Microbiol 171:405-16.

9. Cohen-Bazire G, Kunisawa R. 1963. The fine structure of *Rhodospirillum rubrum*. J Cell Biol 16:401-19.

10. Pfennig N, Lunsdorf H, Suling J, Imhoff JF. 1997. *Rhodospira trueperi* gen. nov., spec. nov., a new phototrophic Proteobacterium of the alpha group. Arch Microbiol 168:39-45.

11. Imhoff JF, Truper HG, Pfennig N. 1984. Rearrangement of the Species and Genera of the Phototrophic Purple Nonsulfur Bacteria. Int J Syst Bacteriol 34:340-343.

12. Boldareva EN, Akimov VN, Boychenko VA, Stadnichuk IN, Moskalenko AA, Makhneva ZK, Gorlenko VM. 2008. *Rhodobaca barguzinensis* sp. nov., a new alkaliphilic purple nonsulfur bacterium isolated from a soda lake of the Barguzin Valley (Buryat Republic, Eastern Siberia). Microbiology 77:206-218.

13. Hiraishi A, Ueda Y. 1994. Intrageneric Structure of the Genus Rhodobacter - Transfer of *Rhodobacter sulfidophilus* and Related Marine Species to the Genus *Rhodovulum* gen. nov. Int J Syst Bacteriol 44:15-23.

14. Conti SF, Hirsch P. 1965. Biology of Budding Bacteria. 3. Fine Structure of *Rhodomicrobium* and *Hyphomicrobium* spp. J Bacteriol 89:503-12.

15. Duchow E, Douglas HC. 1949. *Rhodomicrobium vannielii*, a New Photoheterotrophic Bacterium. J Bacteriol 58:409-16.

16. Hiraishi A, Urata K, Satoh T. 1995. A new genus of marine budding phototrophic bacteria, *Rhodobium* gen. nov., which includes *Rhodobium orientis* sp. nov. and *Rhodobium marinum* comb. nov. Int J Syst Bacteriol 45:226-34.

17. Imhoff JF. 1983. *Rhodopseudomonas marina* sp. nov., a New Marine Phototropic Purple Bacterium. Syst Appl Microbiol 4:512-521.

18. Urdiain M, Lopez-Lopez A, Gonzalo C, Busse HJ, Langer S, Kampfer P, Rossello-Mora R. 2008. Reclassification of *Rhodobium marinum* and *Rhodobium pfennigii* as *Afifella marina* gen. nov. comb. nov. and *Afifella pfennigii* comb. nov., a new genus of photoheterotrophic Alphaproteobacteria and emended descriptions of *Rhodobium*, *Rhodobium orientis* and *Rhodobium gokarnense*. Syst Appl Microbiol 31:339-51.

19. Caumette P, Guyoneaud R, Duran R, Cravo-Laureau C, Matheron R. 2007. *Rhodobium pfennigii* sp. nov., a phototrophic purple non-sulfur bacterium with unusual bacteriochlorophyll a antennae, isolated from a brackish microbial mat on Rangiroa atoll, French Polynesia. Int J Syst Evol Microbiol 57:1250-5.

20. Imhoff JF. 2001. Transfer of *Rhodopseudomonas acidophila* to the new genus *Rhodoblastus* as *Rhodoblastus acidophilus* gen. nov., comb. nov. Int J Syst Evol Microbiol 51:1863-6.

21. Pfennig N. 1969. *Rhodopseudomonas acidophila*, sp. n., a new species of the budding purple nonsulfur bacteria. J Bacteriol 99:597-602.

22. Drews G, Giesbrecht P. 1966. [*Rhodopseudomonas viridis*, n. sp., a newly isolated, obligate phototrophic bacterium]. Arch Mikrobiol 53:255-62.

23. Hiraishi A. 1997. Transfer of the bacteriochlorophyll b-containing phototrophic bacteria *Rhodopseudomonas viridis* and *Rhodopseudomonas sulfoviridis* to the genus *Blastochloris* gen. nov. Int J Syst Bacteriol 47:217-9.

24. Chakravarthy SK, Ramaprasad EV, Shobha E, Sasikala C, Ramana Ch V. 2012. *Rhodoplanes piscinae* sp. nov. isolated from pond water. Int J Syst Evol Microbiol 62:2828-34.

25. Hiraishi A, Ueda Y. 1994. *Rhodoplanes* gen. nov., a New Genus of Phototrophic Bacteria Including *Rhodopseudomonas rosea* as *Rhodoplanes roseus* comb. Nov. and *Rhodoplanes elegans* sp. nov. Int J Syst Bacteriol 44:665-673.

26. Janssen PH, Harfoot CG. 1991. *Rhodopseudomonas rosea* sp. nov., a New Purple Nonsulfur Bacterium. Int J Syst Bacteriol 41:26-30.

27. Whittenbury R, McLee AG. 1967. *Rhodopseudomonas palustris* and *Rh. viridis*--photosynthetic budding bacteria. Arch Mikrobiol 59:324-34.

28. Takaichi S. 2009. Distribution and Biosynthesis of Carotenoids, p 97-117. *In* Hunter CN, Daldal F, Thurnauer MC, Beatty JT (ed), The Purple Phototrophic Bacteria. Springer Netherlands, Dordrecht.

29. Kawasaki H, Hoshino Y, Kuraishi H, Yamasato K. 1992. *Rhodocista centenaria* gen. nov., sp. nov., a Cyst-Forming Anoxygenic Photosynthetic Bacterium and Its Phylogenetic Position in the Proteobacteria Alpha-Group. J Gen Appl Microbiol 38:541-551.
