# Supplementary material for: Restricted Localization of Photosynthetic Intracytoplasmic Membranes (ICMs) in Multiple Genera of Purple Nonsulfur Bacteria
Source: mBio. 2018 Jul 3;9(4):e00780-18. doi: 10.1128/mBio.00780-18 (PMC6030561; doi:10.1128/mBio.00780-18)
Supplement: TABLE S2 [file mbo004183956st2.docx]

**Table S2. Bacterial strains, plasmids, and primers used in this study.**

| **Species, strain or plasmid** | **Description or Sequence (5’-3’)** | **Reference or Purpose** |
| --- | --- | --- |
| ***Rhodopseudomonas palustris* strains** |  |  |
| CGA009 | Wild-type strain; spontaneous Cm^R^ derivative of CGA001 | (1) |
| CGA4030 | CGA009 Δ*crtI*; carotenoid-deficient mutant | This study |
| CGA4040 | CGA009 Δ*bchXYZ*; bacteriochlorophyll-deficient mutant | This study |
| DX-1 | Wild-type isolate | (2) |
| TIE-1 | Wild-type isolate | (3) |
| BisB5 | Wild-type isolate | (4) |
| HAa2 | Wild-type isolate | (4) |
| **Other species** |  |  |
| *Afifella marina* | Type strain, DSM2698 | (5) |
| *Afifella pfennigii* | Type strain, DSM 17143 | (5) |
| *Rhodobacter capsulatus* | Wild-type isolate, ATCC BAA-309 (SB1003) | (6) |
| *Rhodobacter sphaeroides* | Type strain, ATCC 17023 (2.4.1) | (7) |
| *Rhodobium orientis* | Type strain, DSM 11290 | (5) |
| *Rhodoplanes elegans* | Type strain, DSM 11907 (AS130) | (8) |
| *Rhodoplanes piscinae* | Type strain, DSM 19946 (JA266) | (9) |
| *Rhodoplanes roseus* | Type strain, DSM 5909 (941) | (10) |
| *Rhodospirillum centenum* | Wild-type strain, ATCC 51521 (SW) | (11) |
| *Rhodospirillum rubrum* | UR2, Spontaneous Sm^R^ derivative of *R. rubrum* ATCC 11170 | (12) |
| *Rhodoblastus acidophilus* | Type strain, DSM 137 (7050) | (13) |
| *Rhodomicrobium vannielii* | Type strain, ATCC 17100 | (14) |
| *Rhodovulum sulfidophilum* | Type strain, DSM 1374 | (15) |
| **Plasmids** |  |  |
| pJQ200SK | *R. palustris* suicide vector, Gm^r^ | (16) |
| pJQcrtIKO | pJQ200SK with DNA fragments flanking *crtI* fused by PCR to generate unmarked deletion | This study |
| pJQbchXYZKO | pJQ200SK with DNA fragments flanking *bchXYZ* assembled using Gibson Assembly to generate unmarked deletion | This study |
| **Primers** |  |  |
| BL530 | GACTTCTAGAgatcctgctgattcacggcac | *crtI* upstream flanking region; xbaI |
| BL531 | GACTGAGCTCgagcatgggtaccactgtaaca | *crtI* upstream flanking region; sacI |
| BL532 | GACTGAGCTCtgagtctgcaatccgatatgttggc | *crtI* downstream flanking region; sacI |
| BL533 | GACTTCTAGAttcggtttcctggaacgccaa | *crtI* downstream flanking region; xbaI |
| BL557 | ccaccgcggtggcggccgcttggctgcgactgcttatcatgc | *bchX* upstream flanking region |
| BL558 | catcgttacatcagctcggtctccaat | *bchX* upstream flanking region |
| BL559 | accgagctgatgtaacgatgatttgagctagaacaatgc | *bchZ* downstream flanking region |
| BL560 | cctgcagcccgggggatcctcaacgacgatgaagaggaagaag | *bchZ* downstream flanking region |

**Table S2 References**

1. Kim MK, Harwood CS. 1991. Regulation of Benzoate-Coa Ligase in *Rhodopseudomonas palustris*. FEMS Microbiol Lett 83:199-203.

2. Xing D, Zuo Y, Cheng S, Regan JM, Logan BE. 2008. Electricity generation by *Rhodopseudomonas palustris* DX-1. Environ Sci Technol 42:4146-51.

3. Jiao Y, Kappler A, Croal LR, Newman DK. 2005. Isolation and characterization of a genetically tractable photoautotrophic Fe(II)-oxidizing bacterium, *Rhodopseudomonas palustris* strain TIE-1. Appl Environ Microbiol 71:4487-96.

4. Oda Y, Larimer FW, Chain PS, Malfatti S, Shin MV, Vergez LM, Hauser L, Land ML, Braatsch S, Beatty JT, Pelletier DA, Schaefer AL, Harwood CS. 2008. Multiple genome sequences reveal adaptations of a phototrophic bacterium to sediment microenvironments. Proc Natl Acad Sci U S A 105:18543-8.

5. Urdiain M, Lopez-Lopez A, Gonzalo C, Busse HJ, Langer S, Kampfer P, Rossello-Mora R. 2008. Reclassification of *Rhodobium marinum* and *Rhodobium pfennigii* as *Afifella marina* gen. nov. comb. nov. and *Afifella pfennigii* comb. nov., a new genus of photoheterotrophic Alphaproteobacteria and emended descriptions of *Rhodobium*, *Rhodobium orientis* and *Rhodobium gokarnense*. Syst Appl Microbiol 31:339-51.

6. Yen HC, Marrs B. 1976. Map of genes for carotenoid and bacteriochlorophyll biosynthesis in *Rhodopseudomonas capsulata*. J Bacteriol 126:619-29.

7. van Niel CB. 1944. The Culture, General Physiology, Morphology, and Classification of the Non-Sulfur Purple and Brown Bacteria. Bacteriol Rev 8:1-118.

8. Hiraishi A, Ueda Y. 1994. *Rhodoplanes* gen. nov., a New Genus of Phototrophic Bacteria Including *Rhodopseudomonas rosea* as *Rhodoplanes roseus* comb. Nov. and *Rhodoplanes elegans* sp. nov. Int J Syst Bacteriol 44:665-673.

9. Chakravarthy SK, Ramaprasad EV, Shobha E, Sasikala C, Ramana Ch V. 2012. *Rhodoplanes piscinae* sp. nov. isolated from pond water. Int J Syst Evol Microbiol 62:2828-34.

10. Janssen PH, Harfoot CG. 1991. *Rhodopseudomonas rosea* sp. nov., a New Purple Nonsulfur Bacterium. Int J Syst Bacteriol 41:26-30.

11. Lu YK, Marden J, Han M, Swingley WD, Mastrian SD, Chowdhury SR, Hao J, Helmy T, Kim S, Kurdoglu AA, Matthies HJ, Rollo D, Stothard P, Blankenship RE, Bauer CE, Touchman JW. 2010. Metabolic flexibility revealed in the genome of the cyst-forming alpha-1 proteobacterium *Rhodospirillum centenum*. BMC Genomics 11:325.

12. Singer SW, Hirst MB, Ludden PW. 2006. CO-dependent H2 evolution by *Rhodospirillum rubrum*: role of CODH:CooF complex. Biochim Biophys Acta 1757:1582-91.

13. Pfennig N. 1969. *Rhodopseudomonas acidophila*, sp. n., a new species of the budding purple nonsulfur bacteria. J Bacteriol 99:597-602.

14. Duchow E, Douglas HC. 1949. *Rhodomicrobium vannielii*, a New Photoheterotrophic Bacterium. J Bacteriol 58:409-16.

15. Hiraishi A, Ueda Y. 1994. Intrageneric Structure of the Genus *Rhodobacter* - Transfer of *Rhodobacter sulfidophilus* and Related Marine Species to the Genus *Rhodovulum* gen. nov. Int J Syst Bacteriol 44:15-23.

16. Quandt J, Hynes MF. 1993. Versatile suicide vectors which allow direct selection for gene replacement in gram-negative bacteria. Gene 127:15-21.
