# Supplementary material for: Restricted Localization of Photosynthetic Intracytoplasmic Membranes (ICMs) in Multiple Genera of Purple Nonsulfur Bacteria
Source: mBio. 2018 Jul 3;9(4):e00780-18. doi: 10.1128/mBio.00780-18 (PMC6030561; doi:10.1128/mBio.00780-18)
Supplement: TABLE S3 [file mbo004183956st3.docx]

**Table S3. Alternative microscopes and filter sets for visualizing BChl-*a* fluorescence.**

| **Microscopes** | **Filter set** | | **Note** |
| --- | --- | --- | --- |
|  | **Name (source)** | **Excitation center/range; emission center/range (nm)** |  |
| Nikon Eclipse TiE  or  Nikon Eclipse Ti2  (Lumencor Spectra X light source for both) | AT DAPI/UV Long Pass (Chroma Cat # 19000) | 375/28; 435 long pass | Perfect focus system dichroic mirror must be in the out position in order to see autofluorescence |
|  | AT DAPI/Hoechst/Alexa Fluor 350 (Chroma Cat # 39000) | 375/28; 460/50 |  |
|  | AT Aqua Long Pass (Chroma Cat # 19001) | 420/40; 465 long pass |  |
|  | 385 nm LED-DAPI (Chroma, sold specifically through Nikon Product # 96240) | 390/38; 475/90 |  |
|  | ET DAPI 395 (Chroma Cat # 49028) | 395/25; 460/50 |  |

BChl-*a* fluorescence is not detectable using a DAPI/FITC/TRITC/Cy5 Quad filter set (Chroma Cat # 89401) (DAPI excitation 396/30; emission 447/40) regardless of the PFS dichroic mirror position.
